# The roles of NOP56 in cancer and SCA36

**DOI:** 10.3389/pore.2023.1610884

**Published:** 2023-01-19

**Authors:** Shimin Zhao, Dongdong Zhang, Sicheng Liu, Jun Huang

**Affiliations:** ^1^ Department of General Surgery, The Second Affiliated Hospital of Nanchang University, Nanchang, China; ^2^ Jiangxi Province Key Laboratory of Molecular Medicine, The Second Affiliated Hospital of Nanchang University, Nanchang, China

**Keywords:** methylation, NOP56, malignant tumor, box C/D RNP, SCA36

## Abstract

NOP56 is a highly conserved nucleolar protein. Amplification of the intron GGCCTG hexanucleotide repeat sequence of the *NOP56* gene results in spinal cerebellar ataxia type 36 (SCA36). NOP56 contains an N-terminal domain, a coiled-coil domain, and a C-terminal domain. Nucleolar protein NOP56 is significantly abnormally expressed in a number of malignant tumors, and its mechanism is different in different tumors, but its regulatory mechanism in most tumors has not been fully explored. NOP56 promotes tumorigenesis in some cancers and inhibits tumorigenesis in others. In addition, NOP56 is associated with methylation in some tumors, suggesting that NOP56 has the potential to become a tumor-specific marker. This review focuses on the structure, function, related signaling pathways, and role of NOP56 in the progression of various malignancies, and discusses the progression of NOP56 in neurodegenerative and other diseases.

## Introduction

NOP56 is an essential nucleolar protein with a molecular mass of 66 KDa. NOP56 and NOP58 are highly homologous throughout the sequence, with a KKE/D repeat at the carboxyl terminus, and like NOP58, NOP56 is highly conserved from yeast to human (1, 2). NOP56, NOP58, fibrillarin (FBL), SNU13, and more than a dozen small nucleolar RNAs (snoRNAs) are involved in ribosome production in eukaryotes (3). The snoRNAs are divided into three categories: two main categories of snoRNAs are C/D box snoRNAs and H/ACA box RNAs, respectively, and the third category of snoRNAs are small Cajal body specific RNAs (scaRNAs), which have the complex functions of the first two snoRNAs (4). C/D box snoRNA exists in the nucleolus as a stable form of small nucleolar ribonucleoprotein complex (snoRNP) (5). NOP56 has an N-terminal domain (NTD), a coiled-coil domain (CC), and a C-terminal domain (CTD) (6).

In recent years, more and more studies have demonstrated that dysregulation of NOP56 is closely related to the occurrence and development of various malignant tumors. The expression of NOP56 is significantly upregulated in acute myeloid leukemia, diffuse large B-cell lymphoma, and Myc-mutant Burkitt’s lymphoma, and may also be associated with poorer prognosis (7–9). Downregulation of NOP56 expression significantly inhibited the proliferation of KRAS mutant lung, pancreatic and colorectal cancer cells, and NOP56 mRNA levels have been suggested to be a predictive marker of rapamycin sensitivity (IC50) in KRAS mutant cancers (10). The study found that the expansion of the intron GGCCTG hexanucleotide repeat of NOP56 gene will lead to a new phenotype of spinocerebellar ataxias (SCAs), SCA36, in patients with advanced limb ataxia, dystonia and dysarthria (11–15). However, the biological functions and molecular mechanisms of NOP56 in cancer remain largely unexplored. Therefore, in this review, we discuss the structure, function, and knowledge of the NOP56 related signaling pathway, and emphasize the expression and role of NOP56 in various diseases.

## Overview of NOP56

### NOP56 gene and NOP56 protein functional characteristics

The gene encoding NOP56 is located on human chromosome 20p13 and contains 13 exons (16). NOP56 can participate in the processing of rRNA precursors, promote the synthesis of mature rRNA, and impair rRNA biosynthesis when NOP56 is underexpressed (1). snoRD86 in box C/D snoRNA plays a key role in restrictively controlling the level of box C/D snoRNP core protein NOP56, which is very important for ribosome biosynthesis. Alternative splicing on both sides of snoRD86 provides different donors. The donor provided upstream of snoRD86 is spliced to form mRNA encoding NOP56, and the donor provided downstream of snoRD86 is spliced to form the substrate mRNA of the NMD pathway, which is mediated by SMG6 to generate snoRD86-cSPA, which is a lncRNA. The study found that when NOP58 and FBL are depleted, positive feedback promotes NOP56 production and leads to changes in the nucleolar organization. Conversely, when NOP56 and FBL are depleted, there is positive feedback to promote NOP58 production (17). NOP56 appears to be the last core protein to enter the snoRNP complex and this could be decisive for activation of the complex. Moreover, its level of expression plays a key role in the assembly of 60S ribosomal subunit (18). SUMOylation is a post-translational modification of proteins by small ubiquitin analogs (SUMO), and SUMOylation is crucial in ribosome biogenesis. SUMOylation plays a key role in regulating the interaction of NOP58 with snoRNAs, SUMOylated NOP58 has a higher affinity for snoRNAs, and, SUMOylated NOP58 is essential for the localization of C/D box snoRNAs in nucleolus (19). The deubiquitinase USP36 promotes SUMOylation of NOP56 and NOP58 and promotes binding of NOP58 to snoRNA, and knockout of deubiquitinase USP36 significantly inhibits rRNA processing (20). Myc directly impacts RNA Pol I transcription, and it also affects expression of many nucleolar proteins, which include NOP56 (21). Myc has been shown to participate in ribosome biogenesis and protein synthesis by controlling the expression of the target gene NOP56 (22).

### Box C/D snoRNP structural features

**FIGURE 1:**
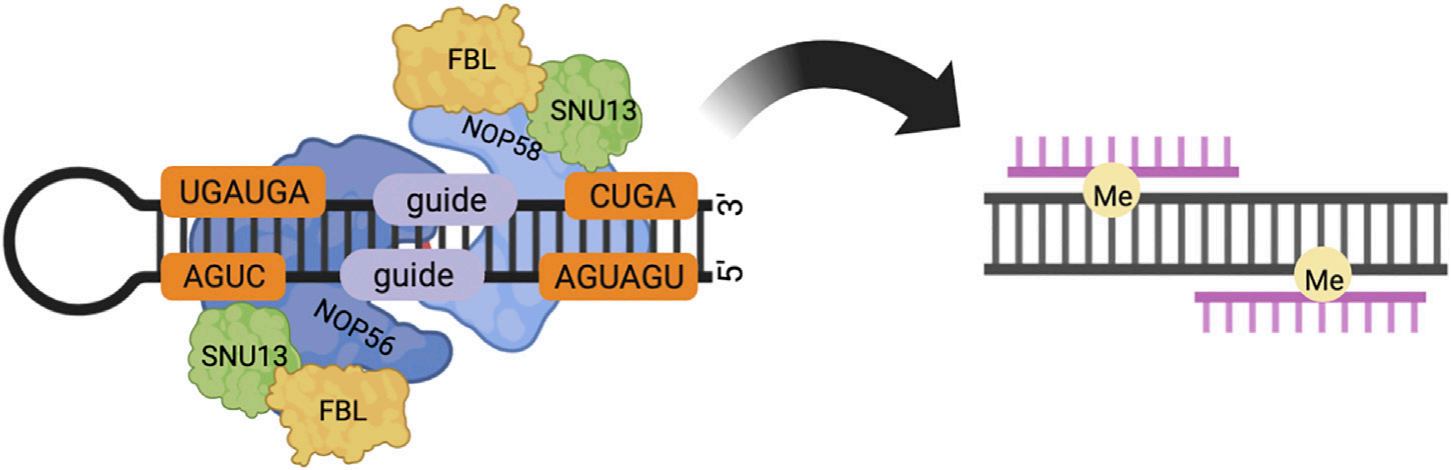
In eukaryotes, box C/D snoRNP consists of core proteins NOP58, NOP56, FBL, SNU13 and box C/D snoRNA. NOP56 and NOP58 bind to C′/D′ and C/D motifs respectively through the C-terminal structural domain. The spacer region between the C/D and C'/D′ motifs contains a guide region, which forms a 10 bp double-stranded RNA with the substrate, and modifies the substrate base corresponding to the fifth base upstream of the D/D′ motif by methylation.

Box C/D snoRNA forms stable RNA-protein complexes (RNPs) with various proteins, which catalyze the site-specific 2′-O-methylation of rRNA, snRNA, and tRNA in an RNA-guided manner (23–26). The box C/D snoRNP in yeast consists of a box C/D snoRNA with NOP56, NOP58, NOP1, and SNU13. The box C/D snoRNP in eukaryotes consists of a box C/D snoRNA with NOP56, NOP58, fibrillarin (FBL, NOP1 in Saccharomyces cerevisiae), and SNU13 (15.5K in Homo sapiens). Box C/D snoRNP in Archaea consists of a box C/D snoRNA with Nop5, fibrillarin, and L7Ae. Fibrillarin contains the well-conserved S-adeninosine-L-methionine (SAM) binding motif and is thus the center of methyltransferase. Fibrillarin has a common structural domain consisting of a mixture of seven central β-sheets and α-sheets (23–25). NOP56 and NOP58 are a pair of homologous proteins, which are also homologous to Nop5 in Archaea, and target the C'/D′ motif inside the box C/D snoRNA and the C/D motif at the end, respectively (26,27). Nop5/56/58 contains three domains, an N-terminal domain (NTD), a coiled-coil domain (CC), and a C-terminal domain (CTD). NOP56 and NOP58 are the core proteins of two of the box C/D snoRNP complexes that interact through the coiled-coil structural domain (28). The C-terminal domain is highly charged and conserved in evolution. In yeast, the CTD is formed by repeated KKD/E sequences and can directly interact with Tgs1p. In mammalian cells, the CTD contains the nucleolar localization signal (NoLS) of box C/D snoRNP, and the CTD of NOP58 can directly bind to TGS1. TGS1-LF (full-length form of the protein, see below) can mask these nucleolar localization signals (1, 29). The two long coils of the coiled-coil domain can take either parallel or intersecting positions. The NTD and the coiled-coil domain form a hinge N, and the CTD and the coiled-coil domain form a hinge C, around which the NTD and CTD can rotate. The box C/D snoRNA has a symmetrical stem-loop structure, including a C-box (RUGAUGA) and a D-box (CUGA) at the end of the snoRNA, and the related C’box and D’box inside (26). After binding to SNU13/L7Ae protein, the C/D or C′/D′ motif forms an acute bend (K-turn or K-loop) structure (30). There are two spacer RNA sequences between the C/D and C'/D′ motifs. The average length of Archaeal spacers is 12–13 nt, and the length of eukaryotic spacers ranges from a few bases to hundreds of bases. The spacer region contains a guide region, which forms a 10 bp double-stranded RNA with the substrate through the principle of complementary base pairing, and the substrate base corresponding to the fifth base upstream of the D/D′ motif is methylated modification (Figure 1). In Archaea, spacers that are too long (more than 14 nt) or too short (less than 12 nt) do not affect box C/D and C'/D′ RNP assembly, but do affect the activity of the target RNA methylation modification of the box C/D and C'/D′ RNP lineage (3, 31, 32).

In Archaea, L7Ae first recognizes box C/D snoRNA, assembles to form L7Ae-C/D RNA complex, and then recruits fibrillarin by binding Nop5 to form L7Ae- C/D RNP complex in which L7Ae recognizes the CTD domain of Nop5 (33–37). In eukaryotes, assembly of the box C/D snoRNP requires the HSP90/R2TP system, and the R2TP complex consists of PIH1D1, RPAP3, and RUVBL1 and RUVBL2. RUVBL1 and RUVBL2 are two highly conserved AAA + ATPases that form a heterohexamer complex. ATP binds to the R2TP complex to release RUVBL1 and RUVBL2 from the R2TP complex and load them onto SNU13 and NOP58. The assembly factors NUFIP, ZNHIT3 and ZNHIT6 bind to it to form a pure protein complex. The complex binds to box C/D snoRNA, recruits FBL and NOP56 and removes NUFIP, ZNHIT3, ZNHIT6, RUVBL1, and RUVBL2, forming box C/D snoRNP particles (18, 38). Box C/D snoRNA has a long GU repeat sequence, and TCAB1 (also known as WRAP53/WDR79) recognizes the long GU repeat sequence. TCAB1 sends a translocation signal to transport box C/D snoRNP particles to Cajal bodies (CBs) (39). TGS1 has two isoforms: a full-length protein called TGS1 long type (TGS1-LF), which is mainly localized in the cytoplasm and CBs. The other is the short type (TGS1-SF), produced by proteolytic cleavage of its N-terminal structural domain, is mainly localized in the CBs and nucleoplasm (40). TGS1-LF binds to the C-terminal structural domains of NOP56 and NOP58, and CRM1 reduces the affinity of TGS1-LF for NOP58. The reduced affinity of TGS1- LF for NOP58 may activate the nucleolus localization signal (NoLS) of NOP58 to facilitate translocation of box C/D snoRNP particles from CBs to the nucleolus (29).

### NOP56 and signaling pathways

NOP56 is located at the key crossroads of many signaling pathways and plays an important role in tumorigenesis and development. Exploring the role of NOP56 in these signaling pathways will help us further understand the mechanism of tumorigenesis and development. The p38 pathway is one of the major mitogen-activated protein kinase (MAPK) signaling pathways and plays an important role in many cellular processes, including inflammation, cell differentiation, cell growth, and cell death (41). In KRAS-mutant NSCLC, inhibition of NOP56 expression resulted in a significant increase in IRE1α, and NOP56 mediated UPR activation of mTOR signaling *via* p38/MAPK (10). NF-κB is a transcription factor in the TLR signaling pathway and plays a key role in the innate immune response. The NF-κB family includes p65 (RelA), NF-εB1 (p50/p105), NF-κB2 (p52/p100), RelB and c-Rel (42). Studies in teleosts reported that overexpression of NOP56 significantly inhibited MyD88-mediated NF-κB signaling pathway and suppressed IL-1β and IL-8 transcription. And the NOSIC domain of NOP56 is critical for the inhibition of MyD88-mediated NF-κB signaling pathway by NOP56 (43). The JAK/STAT signaling pathway is highly conserved from invertebrates to vertebrates and plays an important role in many growth and developmental processes. Studies have shown that dysregulation of the JAK/STAT signaling pathway leads to the development and progression of a variety of malignancies and autoimmune diseases (44). In Drosophila studies, NOP56 has been found to be a functional target of the JAK/STAT signaling pathway, and the JAK/STAT signaling pathway promotes Drosophila neuroepithelial growth through activation of NOP56 (45).

## The expression of NOP56 in cancer

**FIGURE 2:**
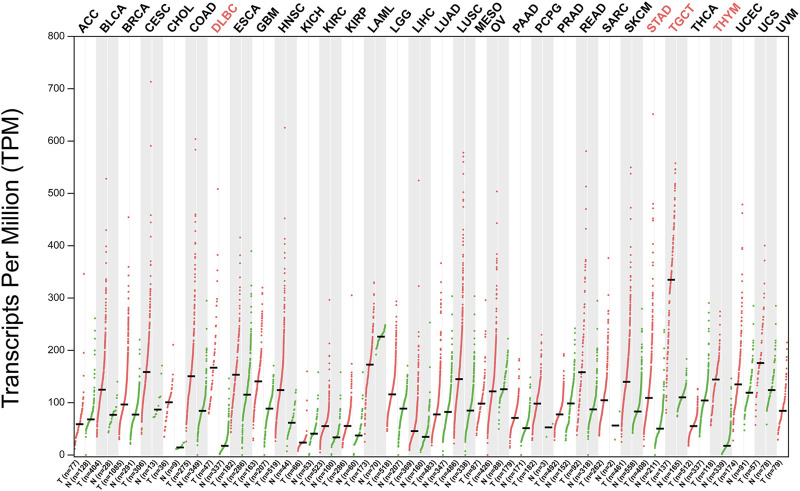
NOP56 expression in cancer. Expression level of NOP56 across 33 TCGA tumors compared to TCGA normal using GEPIA. For each TCGA tumor (red), its matched normal (green) are given; T: tumor; N: normal; Y axis: transcript per million. X axis: number of tumor and normal samples. Acronyms of cancers with statistically significant differences are shown in red. ACC, adrenocortical carcinoma; BLCA, bladder urothelial carcinoma; BRCA, breast invasive carcinoma; CESC, cervical squamous cell carcinoma and endocervical adenocarcinoma; CHOL, cholangiocarcinoma; COAD, colon adenocarcinoma; DLBC, lymphoid neoplasm diffuse large B-cell lymphoma; ESCA, esophageal carcinoma; GBM, glioblastoma multiforme; HNSC, head and neck squamous cell carcinoma; KICH, kidney chromophobe; KIRC, kidney renal clear cell carcinoma; KIRP, kidney renal papillary cell carcinoma; LAML, acute myeloid leukemia; LGG, brain lower grade glioma; LIHC, liver hepatocellular carcinoma; LUAD, lung adenocarcinoma; LUSC, lung squamous cell carcinoma; MESO, mesothelioma; OV, ovarian serous cystadenocarcinoma; PAAD, pancreatic adenocarcinoma; PCPG, pheochromocytoma and paraganglioma; PRAD, prostate adenocarcinoma; READ, rectum adenocarcinoma; SARC, Sarcoma; SKCM, skin cutaneous melanoma; STAD, stomach adenocarcinoma; TGCT, testicular germ cell tumors; THCA, thyroid carcinoma; THYM, thymoma; UCEC, uterine corpus endometrial carcinoma; UCS, uterine carcinosarcoma; UVM, uveal melanoma. Data was downloaded from GEPIA database (http://gepia2.cancer-pku.cn/#analysis).

An increasing number of studies have proved that abnormal expression of NOP56 is closely related to the occurrence and development of various tumors (Figure 2). NOP56 is significantly increased in KRAS-mutant non-small cell lung cancer, KRAS-mutant pancreatic cancer, KRAS-mutant colorectal cancer, Wilms tumor, diffuse large B-cell lymphoma, Myc-mutant Burkitt’s lymphoma, breast cancer, prostate cancer, and acute lymphoblastic leukemia (8, 9, 46–51). Hypermethylation in the NOP56 promoter region in HPV+ cervical cancer leads to downregulation of NOP56 (52). NOP56 promotes the occurrence and development of tumor cells by activating signaling pathways, inhibition of reactive oxygen species production, regulation of tumor-like stem cell pluripotency, and DNA hypermethylation. DNA methylation is the most common epigenetic modification in human tumors, and hypermethylation of gene promoter CpG islands has been associated with inactivation of tumor suppressor genes in tumor cells, particularly in colorectal cancer (53, 54). Therefore, hypermethylation of NOP56 gene has the potential to become a tumor-specific marker in the future. These findings indicate that NOP56 plays diverse roles in tumor progression and the details are specifically summarized in Table 1. NOP56 is one of many methylated genes, and examining the methylation status of genes can help identify tumor-specific markers and therapeutic targets for cancer patients. There are three main methods for genome-wide DNA methylation analysis commonly used today: the first is restriction enzyme-based techniques, the second is affinity enrichment-based techniques, and the third is bisulfite conversion-based methods (55).

**TABLE 1:** The expression and roles of NOP56 in different human cancers.

Cancer type	Levels of NOP56	Function	Relevant targets/mechanisms/signaling pathways	Types of references	Reference:
Non-small cell lung cancer (NSCLC)	Up	Inhibit ROS and reduce sensitivity to chemotherapy drugs	IRE1α, UPR, p38/MAPK/mTOR	Cell culture	(10)
Wilms tumor(WT)	Up	Inhibit tumor cell proliferation, invasion and migration, and promote tumor cell apoptosis	CD24, CD133, KLF4 and SOX2	Cell culture	(47, 48)
Diffuse large B-cell lymphoma (DLBCL)	Up	-	-	Cell culture	(8)
Myc-mutant Burkitt’s lymphoma (BL)	Up	Promote tumor cell proliferation, promote Myc wild-type tumor cell differentiation, and increase tumor volume	-	Cell culture	(9)
Cervical cancer (CCA)	Up	-	E7 protein binds to DNMT1 to enhance the methylation of NOP56 gene promoter, resulting in a decrease in NOP56 expression level	Cell culture	(52)
Hepatocellular carcinoma (HCC)	Up	Significantly correlated with survival prognosis, pathological stage, immune infiltration and tumor progression	NOP56 promoter hypomethylation leads to NOP56 overexpression	Bioinformatics	(66–68)
Acute lymphoblastic leukemia (ALL)	Up	Delayed remission	-	whole body	(7)
Breast cancer	Up	Promotes colony formation and cell growth	-	Cell culture	(49)

### NOP56 and non-small cell lung cancer

*KRAS* is the most frequently mutated oncogene in non-small cell lung cancer (NSCLC) (46). Recent studies have found that the expression of NOP56 in KRAS mutant non-small cell lung cancer cell lines is significantly higher than that in KRAS wild-type non-small cell lung cancer cell lines. In addition, the disease-free survival (DFS) and overall survival (OS) of tumor patients in the NOP56 high expression group were significantly shortened. Knockdown of NOP56 in KRAS-mutant lung cancer cell lines significantly increased reactive oxygen species (ROS) in KRAS-mutant cell lines, resulting in significantly higher levels of apoptosis in KRAS-mutant cell lines than in controls. Inhibition of NOP56 increases IRE1α-mediated UPR activation of mTOR signaling through p38/MAPK, thereby reducing oxidative stress. NOP56 knockdown significantly increases resistance of KRAS mutant cell lines to PI3K/AKT inhibitors (LY294002, AZD5363), anti-mTOR drugs (rapamycin, everolimus), and ER stress inducers (bortezomib and HA15) sensitivity (10). Targeted inhibition of NOP56 expression combined with rapamycin treatment significantly inhibited the growth of KRAS-mutant non-small cell lung cancer (10). Thus, the development of specific NOP56 inhibitors in combination with mTOR inhibitor therapy may be an effective strategy for the treatment of KRAS-mutant lung cancer in the future. In the study of papillary lung adenocarcinoma (PLACs), NOP56 was shown to be a Myc-induced oncogene and NOP56 was significantly upregulated in papillary lung adenocarcinomas (56).

### NOP56 and Wilms tumor

PIWI-interacting RNA (piRNA) is a small non-coding RNA of 24–31 nucleotides (nt) in length found in germ cells and somatic cells (57). Recently, an increasing number of studies have shown that piRNA and PIWI proteins are abnormally expressed in a variety of tumors and can be used as biomarkers for tumor diagnosis and treatment (58). The research shows that NOP56 is highly expressed in Wilms tumor, while the expression level of piRNA NU13 in Wilms tumor (G401) is significantly lower than that in renal tubular epithelial cells (HK2). piRNA NU13 indirectly regulates NOP56, thereby inhibiting the proliferation, invasion, and migration of tumor cells and promoting tumor cell apoptosis (47). Both piRNA MW557525 and NOP56 were significantly highly expressed in piwil2-induced tumor-like stem cells (Piwil2-iCSCs), while inhibition of piRNA MW557525 could increase the expression level of NOP56, and knockdown of NOP56 upregulated the pluripotency markers CD24, CD133, and KLF4 of tumor stem cells and SOX2 expression, and the proliferation ability of tumor-like stem cells in NOP56 silencing group was significantly higher than that in the control group, suggesting that NOP56 inhibits Piwil2-iCSCs proliferation, invasion, migration and stem cell pluripotency, and promotes their apoptosis (48).

### NOP56 and non-Hodgkin lymphoma

Diffuse large B-cell lymphoma (DLBCL) is the most common non-Hodgkin’s lymphoma with high aggressiveness and heterogeneity. The mRNA and protein expression levels of NOP56 are significantly higher in diffuse large B-cell lymphoma than in normal lymphoid tissue. Interestingly, NOP56 expression was significantly downregulated in diffuse large B-cell lymphoma cell lines resistant to adriamycin (Pfeiffer/ADM) compared with diffuse large B-cell lymphoma cell lines (Pfeiffer). However, treatment of adriamycin-resistant diffuse large B-cell lymphoma with paclitaxel was able to significantly reverse this downregulation trend. It indicates that NOP56 and ribosome biosynthesis play a key role in paclitaxel treatment of adriamycin-resistant diffuse large B-cell lymphoma (8). Previous studies have shown that Myc-mutant Burkitt’s lymphoma cells express significantly higher levels of NOP56 compared with Myc wild-type cells. And compared with Myc wild-type cells, the degree of histone acetylation in Myc mutant cells was positively correlated with NOP56 levels. Reduced expression of NOP56 will severely inhibit tumor cell proliferation and promote tumor cell death. Moreover, upregulation of NOP56 expression in Myc wild-type tumor cells promotes Myc wild-type tumor cell differentiation and significantly increases tumor volume (9). This study suggests that NOP56-targeted therapy may be a method to improve treatment efficiency in the molecular targeted therapy of Burkitt’s lymphoma.

### NOP56 and cervical cancer

DNA methylation modification is an epigenetic event that does not lead to permanent changes in DNA sequence. DNA methylation is involved in regulating gene transcription and cell differentiation. DNA hypermethylation is usually associated with gene silencing. DNA demethylation often promotes gene transcription (59). More and more studies have proved that dysregulation of DNA methylation leads to the occurrence and progression of various diseases. Methylation of specific genes will help in the early detection, diagnosis, treatment, and monitoring of the prognosis of cervical cancer patients (60). Cervical cancer is the second leading cause of cancer death in women aged 20–39 years, and HPV is recognized as an important risk factor for cervical cancer, especially HPV16 and HPV18 (61). The E7 protein translated from the HPV16 E7 gene can bind to DNA methyltransferase 1 (DNMT1) to induce methylation of tumor suppressor genes, leading to inactivation of tumor suppressor genes and promoting tumor progression (62–64). The expression of NOP56 in HPV+ cervical cancer cell lines is lower than that in HPV- cervical cancer cell lines. The methylation level of NOP56 in HPV+ cervical cancer cell lines is higher than that in HPV- cervical cancer cell lines. The E7 protein can interact with DNA methyltransferase 1 (DNMT1) and significantly increase the methylation level of the NOP56 gene promoter, which in turn leads to a significant decrease in the expression of the NOP56 gene. Furthermore, treatment of cervical cancer cells with 5′-azacytidine (5-aza), a DNA methyltransferase inhibitor, can significantly reverse the methylation of NOP56 gene promoter, thereby increasing the expression level of NOP56 (52). These further suggest that the NOP56 promoter methylation site may be a therapeutic target for cervical cancer. Currently, there are no studies reporting methods to detect methylation of the NOP56 promoter. However, there are recent studies using liquid biopsy and quantitative methylation-specific PCR analysis to detect circulating tumor DNA and methylation markers (65).

### NOP56 and hepatocellular carcinoma

Based on bioinformatics analysis of gene expression and GCNA-Kpca algorithm analysis, NOP56 and NOP58 are key genes in hepatocellular carcinoma. Moreover, NOP56 is significantly correlated with the survival prognosis, pathological stage, immune infiltration, and tumor progression of patients with hepatocellular carcinoma, and may be used as a target for the diagnosis and treatment of hepatocellular carcinoma in the future. The methylation analysis indicated that the hypomethylation of the NOP56 promoter may lead to the overexpression of NOP56, which proved that expression of NOP56 may be a potential biomarker of hepatocellular carcinoma (66, 67). In hepatocellular carcinoma, NOP56 is considered as a potential immune marker associated with HBV virus, which can be processed and presented by antigen-presenting cells (APCs) to induce immune responses. Moreover, NOP56 is considered as a potential antigen of HBV-related mRNA vaccine for hepatocellular carcinoma (68).

### NOP56 and other tumors

Acute lymphoblastic leukemia (ALL) is the most common malignancy in childhood, accounting for approximately 25% of cancers in children under the age of 15. The most common subtype is B-cell precursor acute lymphoblastic leukemia, with 20% of patients dying of drug resistance or relapse (69). Recent studies have shown that NOP56 and FBL are significantly upregulated in relapsed patients. Even if drug resistance does not occur, high expression of NOP56 will significantly delay the remission of patients (7). Breast cancer is the most common malignant tumor in women, and breast cancer accounts for 30% of female cancers (61). NOP56 significantly promotes colony formation and cell growth in breast cancer tumor cells (49). At the late stage of prostate cancer progression, NOP56 was significantly elevated in both prostate cancer tissue and metastatic tissue (50). The expression of NOP56 in KRAS mutant pancreatic cancer cell lines (MIAPaCa, HPAF-II) and colon cancer cell lines (HCT-116, DLD-1) is significantly higher than that in KRAS wild-type tumor cell lines (46). Studies in endometrial cancer have found that NOP56 correlates with patient survival, suggesting prognostic relevance. However, the difference in NOP56 expression between endometrial cancer and healthy endometrial epithelium was not statistically significant. Using statistical methods, NOP56 was found to be significantly and positively correlated with telomere-associated protein TERF2 (70). In the whole blood mRNA gene expression of metastatic renal cell carcinoma, the increase of NOP56 was statistically significant (71). The mRNA level of NOP56 was significantly upregulated in glioblastoma by bioinformatics analysis in the TCGA-GBM database and Oncomine database (72).

## NOP56 and spinocerebellar ataxia type 36

Spinocerebellar ataxias type 36 (SCA36) is an autosomal dominant heterogenous neurodegenerative disorder with cerebellar ataxia. Patients with cerebellar Purkinje cells and sublingual nucleus motor neurons Absence, resulting in overt ataxia symptoms characterized by loss of balance and rhythm, progressive gait, and limb ataxia (12–14, 73). Expansion of the GGCCTG hexanucleotide repeat in the NOP56 intron results in spinocerebellar ataxia type 36 (SCA36), with marked motor nerve involvement in late stages of SCA36, and a typically suppressed tongue in all affected individuals Atrophy with fascicular contractions results in dysarthria, but the severity varies (13). SCA36 is very rare in mainland China. SCA36 studies in mainland China show that patients in mainland China have a late onset and slow progression, and the main symptoms and signs are ataxia, dysarthria, and hyperreflexia. Most affected patients present with neuropathic hearing loss (74). Today, zebrafish have become an important model for studying neurological diseases, which are highly homologous to human genes, and show a variety of molecules and structures that are homologous to human diseases in the zebrafish central nervous system (75). A study in zebrafish found that NOP56 heterozygous mutants exhibited severe neurodegenerative phenotypes, including cerebellar loss, reduced number of spinal neurons, and motor impairment. NOP56 homozygous mutants show increased apoptosis and early death in zebrafish (76). The study found that the mRNA expression of the ZPLD1 gene was reduced in NOP56 homozygous mutants, and the reduced expression of ZPLD1 was associated with balance dysfunction (77).

## NOP56 and other diseases

Compared with age-matched wild-type mice, transgenic mice of the amyotrophic lateral sclerosis (ALS) model showed a progressive decrease in NOP56 expression levels early in the disease, especially in the large motor neurons of the lumbar and cervical spine (78). Studies have found that hypermethylation in the NOP56 promoter region is not only associated with cancer, but may also be associated with age-related macular degeneration (AMD). Collecting blood samples from AMD patients, the NOP56 promoter region is hypermethylated in AMD, resulting in low expression of NOP56. It is suggested that hypermethylation of NOP56 promoter region may serve as a biomarker for future diagnosis and treatment of age-related macular degeneration (79). NOP56 has also been studied in degenerative disc disease (DDD), through single-cell RNA sequencing (scRNA-seq) reporting and weighted gene co-expression network analysis (WGCNA), found in the cartilage plate, NOP56 is one of the core therapeutic genes in degenerative disc disease (80).

## Conclusion and prospects

In this review, we elucidate the structure and function of NOP56 and box C/D snoRNPs. Studies in various organisms have found that NOP56 affects a variety of signaling pathways and targets, and leads to a variety of epigenetic changes, thereby affecting the biological function of the organism. In recent years, more and more studies have demonstrated that NOP56 is associated with the occurrence and progression of malignant tumors and the treatment of cancer patients. Today, biomarker-based personalized or precision therapy will be a promising approach to improve patient care and prognosis, not only significantly improving patient health, but also reducing treatment-related harm and treatment costs. Although the role and mechanism of NOP56 in the occurrence and development of tumors are still unclear, NOP56 provides a direction for the development and treatment of tumors in the future. Given the previous studies, we have good reasons to believe that NOP56 is a promising therapeutic and prognostic biomarker for future tumor therapy.
